# Diabetes impairs the protective effects of sevoflurane postconditioning in the myocardium subjected to ischemia/ reperfusion injury in rats: important role of Drp1

**DOI:** 10.1186/s12872-021-01906-w

**Published:** 2021-02-16

**Authors:** Jing Yu, Jiandong He, Wenqu Yang, Xiang Wang, Gaoxiang Shi, Yinglei Duan, Hui Wang, Chongfang Han

**Affiliations:** Department of Anesthesiology, Shanxi Bethune Hospital, 99, Longcheng Street, 030032 Taiyuan, China

**Keywords:** Dynamin‐related protein 1, Mitochondrial fission, Diabetes mellitus, Sevoflurane postconditioning, Myocardial ischemia reperfusion injury

## Abstract

**Background:**

Sevoflurane postconditioning (SevP) effectively relieves myocardial ischemia/reperfusion (I/R) injury but performs poorly in the diabetic myocardium. Previous studies have revealed the important role of increased oxidative stress in diabetic tissues. Notably, mitochondrial fission mediated by dynamin-related protein 1 (Drp1) is an upstream pathway of reactive oxygen production. Whether the ineffectiveness of SevP in the diabetic myocardium is related to Drp1-dependent mitochondrial fission remains unknown. This study aimed to explore the important role of Drp1 in the diabetic myocardium and investigate whether Drp1 inhibition could restore the cardioprotective effect of SevP.

**Methods:**

In the first part of the study, adult male Sprague-Dawley rats were divided into 6 groups. Rats in the diabetic groups were fed with high-fat and high-sugar diets for 8 weeks and injected intraperitoneally with streptozotocin (35 mg/kg). Myocardial I/R was induced by 30 min of occlusion of the left anterior descending branch of the coronary artery followed by 120 min of reperfusion. SevP was applied by continuous inhalation of 2.5 % sevoflurane 1 min before reperfusion, which lasted for 10 min. In the second part of the study, we applied mdivi-1 to investigate whether Drp1 inhibition could restore the cardioprotective effect of SevP in the diabetic myocardium. The myocardial infarct size, mitochondrial ultrastructure, apoptosis index, SOD activity, MDA content, and Drp1 expression were detected.

**Results:**

TTC staining and TUNEL results showed that the myocardial infarct size and apoptosis index were increased in the diabetic myocardium. However, SevP significantly alleviated myocardial I/R injury in the normal myocardium but not in the diabetic myocardium. Additionally, we found an elevation in Drp1 expression, accompanied by more severe fission-induced structural damage and oxidative stress in the diabetic myocardium. Interestingly, we discovered that the beneficial effect of SevP was restored by mdivi-1, which significantly suppressed mitochondrial fission and oxidative stress.

**Conclusions:**

Our study demonstrates the crucial role of mitochondrial fission dependent on Drp1 in the diabetic myocardium subjected to I/R, and strongly indicates that Drp1 inhibition may restore the cardioprotective effect of SevP in diabetic rats.

**Supplementary Information:**

The online version contains supplementary material available at 10.1186/s12872-021-01906-w.

## Background

Myocardial ischemia/reperfusion (I/R) injury remains a phenomenon that often occurs in coronary artery bypass surgery, cardiopulmonary resuscitation and organ transplantation, and has been a troubling problem for anaesthesiologists [1]. Sevoflurane application at the initiation of reperfusion has been reported to effectively reduce myocardial I/R injury, a procedure termed as sevoflurane postconditioning (SevP) [2]. However, previous studies have discovered that SevP performs poorly in the diabetic myocardium, and the exact underlying mechanisms have not been elucidated [3].

Recent studies have highlighted the importance of mitochondrial fission in apoptosis that has been proven to be as an upstream pathway of oxygen free radical production [4–6]. Mitochondria are abnormal when the fission process is intensified, promoting the escape of proapoptotic molecules from the mitochondria to the cytoplasm, integral mitochondrial membranes could prevent this process [7]. Mitochondrial fission is mediated by dynamin-related protein 1 (Drp1), which is located in the cytoplasm [8]. Abundant evidence has revealed that the increase of Drp1 expression is closely correlated with diabetes and insulin resistance[9]. Surprisingly, Drp1 inhibition effectively preserves mitochondrial integrity and mitochondrial dysfunction [10]. Leinninger et al. reported increased Drp1 expression, excessive mitochondrial fission, and increased levels of reactive oxygen species in spinal cord dorsal root ganglion cells under hyperglycaemic conditions [11]. The above results suggest that Drp1-related mitochondrial fission and apoptosis directly participate in the pathology of diabetes and its complications. However, whether Drp1 expression is up-regulated in the diabetic myocardium subjected to I/R remains to be further investigated. Thus, we investigated whether Drp1 and its downstream factors are involved in weakening the cardioprotective effect of SevP in the diabetic myocardium against I/R injury.

## Methods

### Animals and groups

Adult male Sprague-Dawley (SD) rats, weighing 220–250 g, were provided by Shanxi Medical University, China. According to Shanxi Medical University Institutional Animal Care, the rats were housed at 22 ± 2℃ and 55 ± 5 % humidity with a cycle of 12/12-h light/dark. The type 2 diabetic model (T2DM) was induced as described previously [12]. The rats were administered a high-fat and high-sugar diet (50 % sucrose, 35 % fat, 15 % protein) for 8 weeks, followed by an intraperitoneal injection of 35 mg/kg of streptozotocin (STZ) (Sigma-Aldrich, St Louis, MO, USA) for 7 days. Rats in the age-matched control group were injected with an equal volume of citrate buffer. All the rats were monitored for blood glucose and body weight every day. Rats with a blood glucose level ≥ 16.7 mmol/L were identified as T2DM rats. All the experimental procedures in this study were performed in accordance with the Regulations for the Administration of Affairs Concerning Experimental Animals approved by the State Council of People’s Republic of China.

**Fig. 1:**
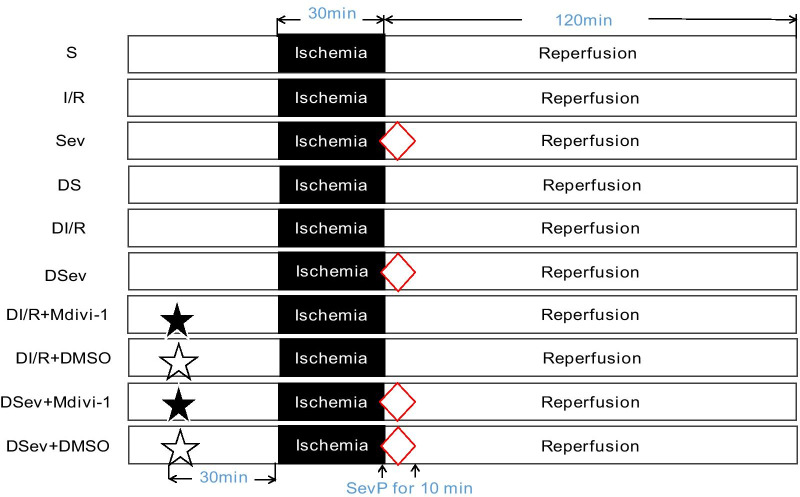
Experimental protocol of each group. S, normal sham group; I/R, normal ischemia/reperfusion group; Sev, normal sevoflurane postconditioning group; DS, diabetic sham group; DI/R, diabetic ischemia/reperfusion group; DSev, diabetic sevoflurane postconditioning group. Upwards arrow and White diamond show that sevoflurane postconditioning was induced 1 min before reperfusion, which lasted for 10 min. Filled star and white star show that Mdivi-1 and its vehicle DMSO were intravenously injected 30 min before ischemia using the same volume

After feeding for one month, the rats were randomly divided into six groups (*n* = 8 each): (1) normal sham group (S); (2) normal ischemia/reperfusion group (I/R); (3) normal sevoflurane postconditioning group (Sev); (4) diabetic sham group (DS); (5) diabetic ischemia/reperfusion group (DI/R); (6) diabetic sevoflurane postconditioning group (DSev). Furthermore, to investigate whether inhibiting Drp1 restored the cardioprotective effects of SevP in diabetic hearts against I/R injury, the following grouping was performed in the second part of our study (*n* = 8 each): (1) DI/R group; (2) DSev group; (3) DI/R + Mdivi-1 group; (4) DI/R + DMSO group; (5) DSev + Mdivi-1 group; (6) DSev + DMSO group. The treatment measures for each group are shown in Fig. 1. Mdivi-l (Sigma, USA; 25 µM) was the selective inhibitor of Drp1, while DMSO was the vehicle control (dissolved in 0.9 % NaCl). The agents were intravenously administered 30 min before ischemia using the same volume.

### Myocardial I/R model

The rats were anesthetised with 25 % urethane 5 ml/kg intraperitoneally. After intubation, they were ventilated artificially using a small animal ventilator (ALC-V8; Shanghai Oort Biotechnology Co., Ltd.). During thoracotomy, the left anterior descending coronary artery (LAD) was occluded. Following 30 min of ischemia, reperfusion was performed for 120 min. The S groups received thoracotomy and threading only, without LAD ligation. The I/R groups were induced by 30 min of ischemia and 120 min of reperfusion. The SevP groups were treated with continuous inhalation of 2.5 % sevoflurane 1 min before reperfusion, which lasted for 10 min. The experimental groups and treatments are shown in Fig. 1. Because the rats were under deep anaesthesia without consciousness, when the hearts were removed from their bodies, the blood circulation stopped, and a straight line was shown on ECG, signifying they were dead under anaesthesia without pain.

### Myocardial infarct size and apoptosis measurement

The LAD was occluded again at the same position after 120 min of reperfusion, followed by an injection of 3ml of Evans blue (Sigma, USA) dye into the aorta. Next, the hearts were removed and frozen in a refrigerator at -20℃ for 20 min. Subsequently, the tissues of the left ventricle (LV) were cut into five 3-mm-thick slices and incubated in a TTC (Sigma, USA) phosphoric acid buffer solution for 15 minutes. The ischemic myocardium, also called the area-at-risk (AAR), was stained red with TTC, while the infarct area remained pale. These slices of LV tissue were dried by filter paper and weighed using an electronic balance. Next, the infarct area and AAR tissue were separated and weighed. The percentage values of the infarct size (IS) to AAR (IS/AAR) and AAR to LV (AAR/LV) were calculated.

 Myocardial tissue slices of 4µm thickness embedded in paraffin were used to determine myocardial apoptosis using a transferase-mediated dUTP-biotin nick end labelling (TUNEL) kit (Wuhan Boster Biological Engineering Co., Ltd, China) according to the manufacturer’s instructions. Five fields of each slice(magnification, 40×) were randomly chosen using a digital pathology scanner (Aperio, USA). The nuclei of apoptotic cells stained brown, while normal nuclei stained blue. The apoptosis index (AI) was calculated as the percentage of positive nuclei to the total number of nuclei.

### Malondialdehyde (MDA) and superoxide dismutase(SOD) measurement

The vitality of SOD was determined using a xanthine oxidase detection kit (Nanjing Jiancheng Bioengineering Institute, China). The MDA content was determined using a thiobarbituric acid detection kit (Nanjing Jiancheng Bioengineering Institute, China ).

### Creatine kinase-MB (CK-MB) and troponin I (TnI) measurement

 The levels of plasma CK-MB and TnI were measured by enzyme-linked immunosorbent assay (ELISA) using a detection kit (Wuhan Boster Biological Engineering Co., Ltd, China) and an Ultra Troponin I detection kit (Nanjing Jiancheng Bioengineering Institute, China) respectively, according to the manufacturer’s instructions.

### Myocardial histopathology and mitochondrial ultrastructure

Myocardial tissue samples of 4-µm thickness embedded in paraffin were stained with hematoxylin and eosin (HE) to further observe the histopathological changes. Myocardial tissues (1-mm^3^ pieces) were fixed in glutaraldehyde phosphate buffer and osmium tetroxide. After being rinsed, dehydrated, embedded and sliced, uranyl acetate and lead citrate double staining was performed. The samples were then examined by transmission electron microscopy (JEM-2100; Japan).

### Determination of Drp1 expression

 Myocardial tissue samples of 4-µm thickness fixed in paraformaldehyde were stained with a rabbit anti-rat polyclonal antibody against Drp1 (1:250; Beijing Biosynthesis Biotechnology Co., Ltd.) followed by goat anti-rabbit IgG secondary antibody(1:250; Beijing Biosynthesis Biotechnology Co., Ltd.) and staining with DAB and haematoxylin. Finally, these slices were observed using a digital pathology scanner. Positive expression of Drp1 was represented by brown granules in the cytoplasm, whose mean optical density (OD) was measured using a BI-2000 medical image analysis system.

Total RNA was extracted from the frozen myocardial tissues using a total RNA extraction kit (Shanghai Biological Technology Co., Ltd). RNA was reverse transcribed to complementary DNA(cDNA) using TaqMan Reverse Transcription Reagents. Next, the Drp1 gene was amplified. The primer sequence and amplified fragment length were as follows: rat Drp1 forward: 5’-ATGCCTGTGGGCTAATGAAC-3’; reverse: 5’-CTCCAATTCGACCACCATCT-3’; product length: 168 bp; rat β-Actin forward: 5’-GTCAGG TCATCAC TATCGGCAAT-3’; reverse: 5’-AGAGGTCTTTACG GATGTCAACGT-3’; product length: 147 bp. β-actin was used as a control, and the expression of Drp1 mRNA was derived using 2^−ΔΔCT^ calculations.

### Statistical analysis

The data are presented as mean ± SD. All the analyses were performed using SPSS 19.0 statistical software (SPSS, USA). For comparisons between multiple groups, one-way ANOVA was used, followed by the Student-Newman -Keuls multiple comparison test. *P* < 0.05 (two-tailed) was considered statistically significant.

## Results

### The cardioprotective effect of SevP is attenuated in T2DM rats

**Fig. 2:**
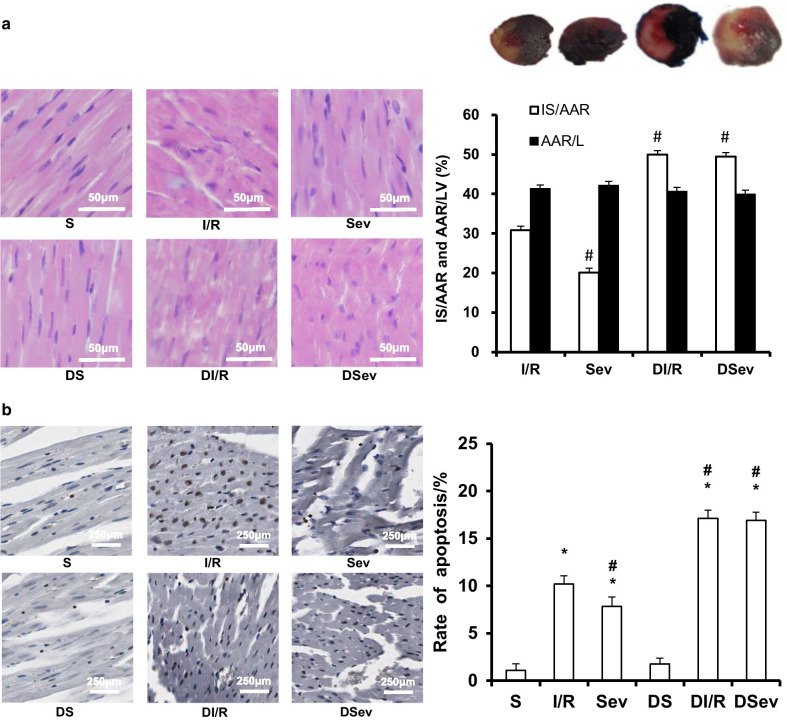
Cardioprotective effect of SevP was attenuated in T2DM rats. **a** Pathological changes of myocardium by HE staining and infarct size expressed as a percentage of IS/AAR (magnification ×400; scale bar: 50 µm; *n* = 3 per group ). **b** Representative images of TUNEL staining and quantitative analysis of TUNEL-positive cell in each group (magnification ×40, scale bar: 250 µm, n = 5 per group). TUNEL-positive cell indicated brown apoptotic nuclei. *P = 0.001 vs. S group; ^#^*P* = 0.001 vs. I/R group

First, we established a T2DM rat model, characterized by a 3.2-fold increase in plasma glucose and an approximately 22 % decrease in body weight compared with normal rats at 4 weeks after STZ injection (Additional file 1: Table 1, *P* < 0.0001). To observe the pathological changes induced by I/R injury, we also performed HE staining (Fig. 2a). Regularly arranged myocardial fibers and intact nuclear membranes were observed in the sham groups, without swelling and inflammatory cell infiltration. However, pathological injury was found in the I/R groups, accompanied by irregular fiber arrangement, inflammatory cell infiltration and broken nuclear membranes. SevP significantly alleviated the pathological injury in the normal groups but not in the diabetic groups, demonstrating more disorganized myocardial fibers and serious inflammatory cell infiltration.

To confirm the cardioprotective effects of SevP against I/R injury, we measured IS and AI. No infarct or ischemic areas were detected in the two sham groups (Fig. 2a). The size of the area at risk (AAR/LV) was similar among the other four groups (*P* = 0.222). SevP remarkably decreased the IS percentage(IS/AAR) by approximately 35 % in normal rats (*P* < 0.0001). However, its cardioprotective effect was absent in the DSev group because the value of IS/AAR was similar to that of the DI/R group (*P* = 0.437). Consistent with the above results, SevP significantly reduced the AI value by 23 % in normal rats (*P* < 0.0001), showing fewer TUNEL-positive cardiomyocytes in the Sev group (Fig. 2b). However, diabetes abolished this effect, because no significant difference was detected in AI between the DI/R and DSev groups (*P* = 0.265).

### SevP decreases myocardial oxidative stress in normal rats but not in diabetic rats

**Fig. 3:**
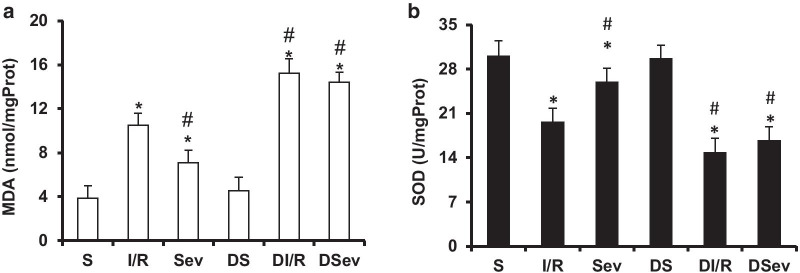
SevP decreased myocardial oxidative stress in normal rats but not in diabetic rats. **a** Measurement of MDA levels assessed by a detection kit. **b** Measurement of total SOD activity assessed by a detection kit. *n* = 8 per group. *P: 0.001 versus. S group; ^#^*P*: 0.001 versus I/R group

Previous studies have confirmed the importance of mitochondrial fission in apoptosis that has also been proven to be an upstream pathway of oxygen free radical production. Therefore, we examined the myocardial oxidative stress response in diabetic rats. SOD activity and the MDA content were chosen to reflect mycardial oxidative stress. SOD is an important enzyme that mainly scavenges reactive oxygen in the body, while MDA is the final product of peroxidation in biological lipid membranes. We detected a significant increase (approximately about 1.45-fold) in MDA levels (*P* < 0.0001) and a significant decrease (approximately 25 %) in SOD activity (*P* < 0.0001) in the DI/R group compared with that in the I/R group (Fig. 3). The results indicated that myocardial oxidative stress induced by I/R obviously deteriorated in diabetic rats. Notably, SevP remarkably reduced the MDA levels by 32 % (*P* < 0.0001) and increased SOD activity by 1.32-fold (*P* < 0.0001). However, no significant difference was found in the MDA levels or SOD activity between the DI/R and DSev groups (*P* = 0.072 and *P* = 0.065, respectively), demonstrating that SevP could not preserve the diabetic heart from oxidative stress injury.

### Excessive mitochondrial fission and increased Drp1 expression are shown in diabetic rats

**Fig. 4:**
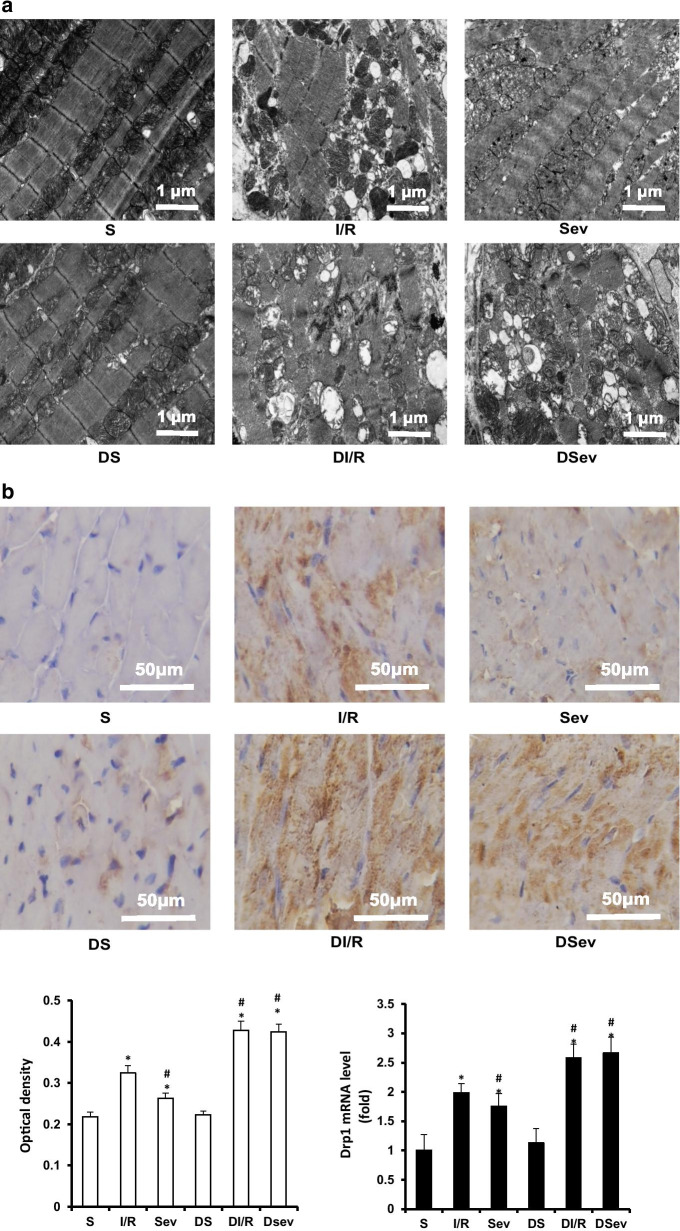
Excessive mitochondrial fission and increased Drp1 expression were shown in diabetic rats. **a** Representative electron microscopic images of mitochondrial ultrastructure of myocardium subjected to ischemia/reperfusion (magnification ×15,000, scale bar: 1µm). More broken and fragmented mitochondria were shown in group DI/R and Dsev. **b** Representative images of Drp1 protein stained by immunohistochemistry, quantitative analysis of Drp1 protein measured by optical density and Drp1 mRNA level (magnification ×400, scale bar: 50 µm). Brown granules indicate positive expression of Drp1. Five fields of sections from left ventricles in each group were randomly selected. Real Time PCR measurement Drp1 transcripts normalized to β-actin transcript was conducted on samples extracted from the left ventricles of rats. *n* = 5 per group.*P: 0.001 vs. S group; ^#^*P*: 0.001 vs. I/R group

We focused on the role of mitochondrial fission in diabetes-weakening cardioprotection induced by SevP. First, we examined the ultrastructural changes in mitochondria by electron microscopy. More swelling and dissolved and fragmented mitochondria were observed in the I/R groups (Fig. 4a). However, SevP alleviated this ultrastructural damage in normal rats, exhibiting intact mitochondrial membranes and orderly arranged mitochondrial cristae. The DI/R and DSev groups showed similar myocardial ultrastructural damage with severe swelling and fragmented mitochondria, accompanied by destroyed cristae, which were hard to discern, suggesting severe ultrastructural damage of mitochondria induced by excessive fission.

Previous studies have revealed that the mitochondrial fission process is mediated by Drp1. Therefore, we assessed the protein and mRNA levels of Drp1. Immunohistochemical staining of Drp1 indicated by brown granules became detectable and evident in the I/R group, while fewer brown granules were detected in the DSev group (Fig. 4b). Drp1 staining was similar between the DI/R and DSev groups, showing more and widely distributed brown granules than the I/R group. In quantitative analysis, we found a significant decrease of approximately 20 % induced by SevP in the optical density (OD) of Drp1 (*P* < 0.0001). Additionally, diabetes treatment obviously intensified the expression of Drp1 protein by 1.34-fold (*P* < 0.0001). However, SevP showed no effect on Drp1 protein expression in diabetic rats subjected to myocardial I/R injury (*P* = 0.389). Our gene expression analysis of Drp1 using quantitative real-time PCR agreed with our immunohistochemistry findings, which showed a decrease of 28 % in the Sev group compared with that in the I/R group (*P* < 0.0001). Additionally, there was an approximately 1.35-fold increase in Drp1 mRNA transcription in the DI/R group compared with that in the I/R group (*P* < 0.0001). The abovementioned results suggest that mitochondrial fission is distinctly increased in diabetic myocardium subjected to I/R, which may subsequently impair mitochondrial morphology and cell function.

### The cardioprotective effect of SevP is restored by the Drp1 inhibitior mdivi-1 in diabetic rats subjected to myocardial I/R injury

**Fig. 5:**
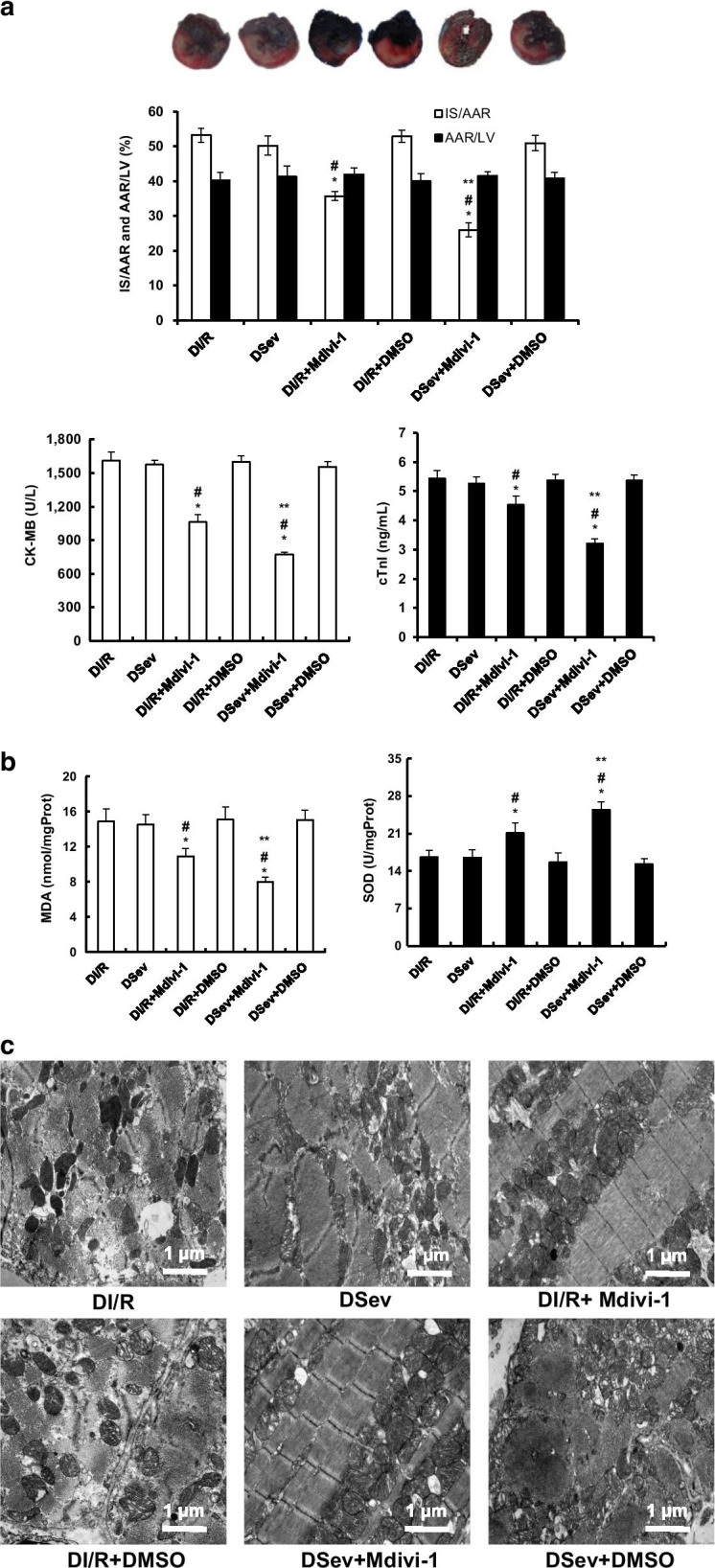
Cardioprotective effect of SevP was restored by Drp1 inhibitior mdivi-1 in diabetic rats subjected to myocardial I/R injury. **a** Infarct size expressed as a percentage of IS/AAR and plasma levels of CK-MB and cTnI assessed by ELISA kit. **b** MDA levels and total SOD activity assessed by detection kits. **c** Representative electron microscopic images of mitochondrial ultrastructure of myocardium subjected to ischemia/reperfusion (magnification ×15,000, scale bar: 1µm). *n* = 8 per group. *P: 0.001 versus DI/R group; ^#^*P*: 0.001 versus DSev group; **P: 0.001 versus DI/R + Mdivi-1group

Drp1 plays an important role in maintaining normal mitochondrial morphology and cell function. Next, we aimed to investigate whether Drp1 inhibition could preserve mitochondrial structure and restore the cardioprotective effect of SevP in diabetic rats. Mdivi-1, which is a selective inhibitor of Drp1, was used in the experiment. Additionally, dephosphorylation of Drp1-S637 (serine 637) results in Drp1 mitochondrial translocation and increased mitochondrial fission, which can be prevented by Mdivi-1 [13]. Additionally, the GTPase activity of Drp1 is inhibited, preventing Drp1 from being located on the mitochondrial outer membrane and further mitochondrial fission[14]. Mdivi-1 was used in our study at a dose of 25 µM according to previous studies to inhibit GTPase activity of Drp1 and effectively preserve mitochondrial morphology [15]. Our pharmacological data strongly suggest a central role for Drp1 inhibition in restoring the cardioprotective effect of SevP. The IS/AAR values in the DSev + mdivi-1 group were significantly decreased by approximately 48 % (*P* < 0.0001) compared with those in the DSev group (Fig. 5a). The cardiac enzymes CK-MB and cTnI were the markers of cardiomyocyte injury, and we found significant decreases of approximately 50 % (*P* < 0.0001) and 40 % (*P* < 0.0001) in CK-MB and cTnI activity, respectively (Fig. 5a).

To validate that mitochondrial fission acts as an upstream pathway of oxidative stress under diabetic conditions, we next determined the levels of SOD and MDA. Mdivi-1 administration markedly reduced oxidative stress in the DSev + mdivi-1 group compared with DSev group, manifested as an increase of 1.48-fold (*P* < 0.0001) and a reduction of 48 % (*P* < 0.0001) in SOD activity and MDA levels respectively, (Fig. 5b). Therefore, the above data suggest that Drp1 is a likely upstream signalling molecule that controls oxidative stress and apoptosis under diabetic conditions. Additionally, transmission electron microscopy showed that mdivi-1 significantly suppressed mitochondrial fission and protected the mitochondrial ultrastructure from diabetes-induced deleterious effects, presenting as intact mitochondrial membranes and tubular and highly interconnected mitochondrial networks, accompanied by orderly arranged mitochondrial cristae (Fig. 5c). Taken together, these results suggest that Drp1 inhibition effectively restores the cardioprotective effect of SevP against diabetic myocardial I/R injury.

## Discussion

Diabetes is one of the most relevant risk factors for cardiovascular disease, particularly coronary heart disease. Some promising therapeutic strategies, including pre-, post-, remote ischemic or pharmacological conditioning, are ineffective in alleviating diabetic myocardial I/R injury [16]. Previous studies have concluded that SevP can protect against I/R injury. However, in our study, we proved that SevP failed to protect the diabetic heart from I/R injury, showing an increased myocardial infarct size and apoptosis in diabetic rats. Little is known about why SevP loses the ability to protect against I/R injury under diabetic conditions. In the present study, we identified that more fragmented and damaged mitochondria in diabetic rats subjected to myocardial I/R injury. Some research has reported that Drp1 participates in regulating the mitochondrial fission process. Importantly, experimental evidence showed that mdivi-1, a selective inhibitor of Drp1, significantly restored the cardioprotective effect of SevP in diabetic rats subjected to myocardial I/R injury. Therefore, inhibition of mitochondrial fission, such as Drp1 inhibition, may represent a promising therapeutic target to restore the cardioprotective effects of SevP to treat myocardial I/R injury in diabetic rats. Myocardial I/R models were generated using the filament model of myocardial I/R injury in which I/R injury might be much more common than that in human myocardial I/R injury. Therefore, the impressive efficacy of regaining the cardioprotective effect of SevP by inhibiting mitochondrial fission that would probably convert to myocardial I/R injury in diabetic patients still requires further study in the future.

Oxidative stress is closely associated with diabetes and its complications, manifested as excessive production of reactive oxide species (ROS) and the depletion of antioxidant defence enzymes [17]. Excessive ROS can damage the bilayer membranes of cells and mitochondria, causing lipid peroxidative reactions of membranes, which are considered a primary mechanism responsible for mitochondrial and cell dysfunction, particularly apoptosis [18]. Excessive oxidative stress under diabetic conditions is a causative factor that accentuates myocardial injury and reduces the heart sensitivity to the cardioprotection of isoflurane, which can be restored by N-acetylcysteine, a scavenger for ROS [19]. In the present study, we explored SOD enzyme activity and MDA levels, which reflected antioxidant systems and lipid peroxidation degree, respectively. Our data demonstrated a decline in SOD activity together with an increase in MDA levels in diabetic myocardium subjected to I/R. Additionally, SevP significantly increased the activity of SOD and decreased the levels of MDA in normal hearts, whereas the abovementioned effects were absent in diabetic rats. However, the inhibiton of ROS production using an inhibitor of Drp1 restored the protective effect of SevP against myocardial I/R injury in diabetic rats. These findings indicate that targeted regulation that modulates ways of ROS production may represent a promising strategy to restore the protective effect of SevP against diabetic myocardial I/R injury.

Mitochondria are major organelles producing ROS. Notably, mitochondrial fission acts as an upstream pathway of ROS production [5, 6]. Excessive mitochondrial fission leads to fragmented mitochondrial membranes and an impaired respiratory chain resulting in the opening of the mitochondrial permeability transition pore (mPTP) and ATP production and increasing ROS production and cell apoptosis [20]. Previous studies have reported that excessive mitochondrial fission regulated by Drp1 is associated with the apoptosis of various cell types under diabetic conditions, including dorsal root ganglion neurons, endothelial cells, and islet cells [11, 21, 22]. Given that mitochondrial fission is aggravated in tissues influenced by diabetes, we hypothesized that a Drp1-dependent mechanism involved in mitochondrial fission played an important role in the insensitivity of the diabetic myocardium to the cardioprotective effects of SevP. Therefore, we explored whether the mitochondrial morphology and Drp1 expression were altered in the diabetic myocardium in rats. Interestingly, our data demonstrated that the expression of Drp1 protein increased, and smaller and fragmented mitochondria with serious structural damage were observed in diabetic rats. SevP failed to preserve the mitochondria from I/R injury in normal rats but not in diabetic rats.

We next targeted the role of Drp1 in the diabetic myocardium subjected to I/R injury by using its inhibitor mdivi-1. Consistent with our hypothesis, the absence of SevP protection in diabetic hearts was reversed by Drp1 inhibition, as evidenced by both a decreased infarct size and reduced levels of CK-MB and TnI in diabetic rats. Additionally, mdivi-1 administration depleted the oxidative stress level and improved mitochondrial morphology, as shown by intact membrane and cristae structures. Furthermore, mdivi-1 together with SevP treatment significantly protected against myocardial I/R injury in diabetic rats, implying the crucial role of Drp1 inhibition in SevP-mediated cardioprotection. However, we cannot conclude that the upregulated expression of Drp1 is the only mechanism responsible for the loss of SevP protection in the diabetic myocardium. Notably, mitochondrial fission mediated by Drp1 is regulated by various posttranslational mechanisms, including ubiquitination, phosphorylation, and S-nitrosylation [23–25]. Additionally, whether a direct link exists between mitochondrial fission and these signalling pathways requires further study.

## Conclusions

In summary, our study demonstrated the ineffectiveness of SevP in relieving myocardial I/R injury in diabetic rats, a finding possibly associated with increased Drp1 expression and mitochondrial fission in the diabetic myocardium, whereas the cardioprotective effects of SevP could be restored by Drp1 inhibition. The results provide novel insights into the crucial role of mitochondrial fission in explaining the insensitivity of diabetic myocardial I/R injury to SevP. Therefore, targeting Drp1 and/or mitochondrial fission may present promising strategies to prevent and treat diabetic myocardial I/R injury.

## Supplementary Information

**Additional file 1**. Changes in plasma glucose and body weight of normal and diabetic groups.

## Data Availability

The dataset used and/or analyzed during the current study is available from the corresponding author on reasonable request.

## Abbreviations

SevP: Sevoflurane postconditioning
I/R: Ischemia/reperfusion
Drp1: Dynamin-related protein 1
SD: Sprague-Dawley
STZ: Streptozotocin
T2DM: Type 2 diabetic model
LAD: Left anterior descending coronary artery
LV: Left ventricle
AAR: Area-at-risk
IS: Infarct size
TUNEL: Transferase-mediated dUTP-biotin nick end labeling
AI: Apoptosis index
CK-MB: Creatine kinase-MB
TnI: Troponin I
MDA: Malondialdehyde
SOD: Superoxide dismutase
HE: Hematoxylin and eosin
OD: Optical density
ROS: Reactive oxide species
mPTP: Mitochondrial permeability transition pore
