# Supplementary material for: Diabetes impairs the protective effects of sevoflurane postconditioning in the myocardium subjected to ischemia/ reperfusion injury in rats: important role of Drp1
Source: BMC Cardiovasc Disord. 2021 Feb 16;21:96. doi: 10.1186/s12872-021-01906-w (PMC7885510; doi:10.1186/s12872-021-01906-w)
Supplement: Supplementary file 1 — Additional file 1. Changes in plasma glucose and body weight of normal and diabetic groups. [file 12872_2021_1906_MOESM1_ESM.docx]

**Table1** Changes in plasma glucose and body weight of normal and diabetic groups

| Group | n | Plasma glucose (mmol/L) | | | | | Body weight (g) | | | | |
| --- | --- | --- | --- | --- | --- | --- | --- | --- | --- | --- | --- |
|  |  | base | 1w | 2w | 3w | 4w | base | 1w | 2w | 3w | 4w |
| Normal | 24 | 6.0±0.8 | 5.8±0.9 | 6.0±0.7 | 6.1±0.9 | 6.4±1.0 | 248±9 | 249±10 | 256±10 | 268±9 | 274±7 |
| Diabetic | 24 | 6.0±0.7 | 26.3±1.4^*^ | 25.7±1.4^*^ | 26.9±1.2^*^ | 27.4±1.3^*^ | 250±10 | 241±10^*^ | 225±8^*^ | 220±11^*^ | 215±10^*^ |

All values are expressed as mean±S.D. Plasma glucose and body weight were measured every day before the establishment of myocardial I/R model. ^*^*P*< 0.0001 versus normal rats at the time of 1, 2, 3, 4 weeks after STZ injection.
